# The little brain supervises learning in the big brain

**DOI:** 10.7554/eLife.109869

**Published:** 2025-12-11

**Authors:** Martha Garcia, Mark Wagner

**Affiliations:** 1 National Institutes of Neurological Disorders and Stroke, National Institutes of Health Bethesda United States

**Keywords:** experience-dependent plasticity, instructive signal, neocortex, pyramidal cell, interneuron, zona incerta, Mouse

## Abstract

Experiments on mice reveal how the cerebellum modulates plasticity in the somatosensory cortex.

**Related research article** Silbaugh A, Koster KP, Hansel C. 2025. Cerebellar climbing fibers impact experience-dependent plasticity in the mouse primary somatosensory cortex. *eLife* **14**:RP109183. doi: 10.7554/eLife.109183.

Countless everyday tasks that seem effortless to many of us are orchestrated by complex networks in the brain. Try to keep track of a moving object – how does your brain keep your eyes stable? Try putting together a sentence – how does your brain keep track of the grammar, syntax, or rhythm of your speech? The cerebral cortex (the outer layer of the brain) has a crucial role in enabling us to perform these tasks, with different areas being responsible for controlling different processes. However, decades of research have taught us that the cerebral cortex has a close ally, the cerebellum, which is located at the back of the head and contains a staggering 80% of the brain’s neurons (Herculano-Houzel, 2010).

The cerebellum (which is Latin for ‘little brain’) is the area of the brain in charge of fine motor coordination and balance, and it keeps our movements smooth and stable by predicting what our next action will be (Wolpert et al., 1998). The cerebellum also has a crucial role in cognition and language processing, and researchers now think that it makes predictions about cognition and emotion, just as it does for movement (Sokolov et al., 2017).

Understanding how the cerebellum contributes to high-level functions involves integrating it into incredibly complex systems that comprise nearly the entire brain. There are multiple areas of the brain connecting the cerebral cortex and the cerebellum, and they exchange information in ways that we are only beginning to understand. For example, we now know that neurons in the cerebellum called climbing fibers respond to events that are salient, novel or rewarding (Hull, 2020). These neurons help shape the cerebellar output that is then sent back to many areas of the brain and body, especially the cerebral cortex (Novello et al., 2024).

Now, in eLife, Abby Silbaugh, Kevin Koster and Christian Hansel from the University of Chicago report the results of experiments on mice which show that climbing fibers can modulate plasticity in the somatosensory cortex, a part of the cerebral cortex that receives and processes sensory input (Silbaugh et al., 2025). Since mice rely heavily on sensing with their whiskers for navigation, a large area of the somatosensory cortex is dedicated to the whiskers. Moreover, this region of the brain is highly plastic – meaning it is easily reshaped by experience, for example losing a whisker.

Silbaugh et al. recorded the activity of cortical pyramidal neurons, which are the principal output neurons in the somatosensory cortex, before and after they used puffs of air to stimulate the whiskers of the mice (Figure 1). Stimulating the whiskers in this way mimics what happens when mice use their whiskers to navigate, and induces experience-dependent plasticity in the somatosensory cortex. Normally, the pyramidal neurons generated larger responses after whisker stimulation. However, when the researchers used optogenetic techniques to selectively stimulate climbing fibers, there was no increase in the activity of the pyramidal neurons, which suggests that the cerebellum can suppress adaptive changes in the cortical circuits that process whisker sensation.

**Figure 1.**
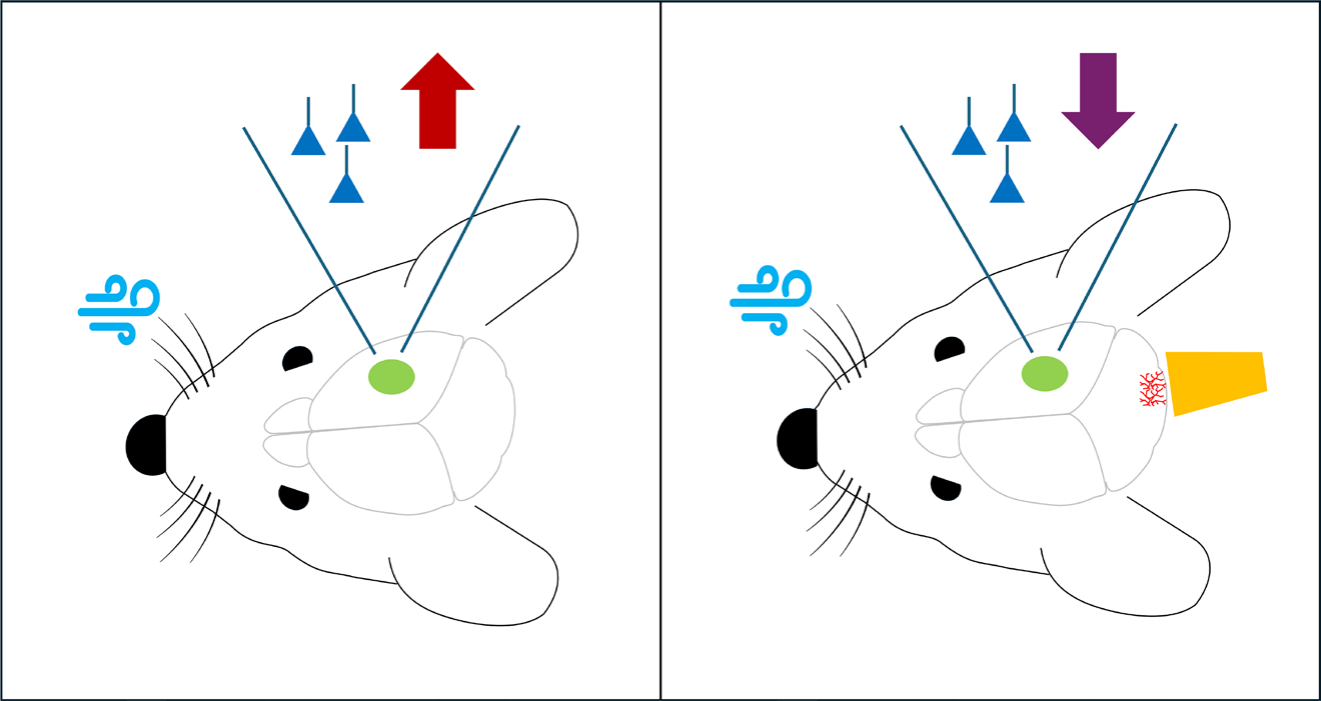
Stimulation of climbing fibers in the cerebellum blocks experience-dependent plasticity in the somatosensory cortex of mice. Left panel: When puffs of air (pale blue) are used to stimulate the whiskers of mice in a way that mimics what happens when mice use their whiskers to navigate, there is an increase (red arrow) in the activity of the pyramidal neurons (dark blue) in the somatosensory cortex (green), which induces experience-dependent plasticity. Right panel: However, when optogenetic techniques are used to stimulate climbing fibers (bright red) in the cerebellum at the same time as the whiskers are being stimulated, there is no increase in the activity of the neurons in the somatosensory cortex, and hence experience-dependent plasticity is blocked (purple arrow). To stimulate the climbing fibers, Silbaugh et al. used brief laser pulses (orange) to trigger a light-sensitive protein called channelrhodopsin that had been introduced into the fibers.

Image credit: Martha Garcia-Garcia and Mark Wagner.

In subsequent experiments, Silbaugh et al. identified a cortical circuit and pathway through which the cerebellum can modulate plasticity in the somatosensory cortex. Naturally, they studied cortical interneurons, which play a crucial role in shaping sensory input as they control information flow to pyramidal neurons. Cortical interneurons also receive long-range inputs from other regions of the cortex, as well as from the thalamus, which is a critical node that relays and processes information from the cerebellum to the cerebral cortex. Silbaugh et al. identified a pathway through an area called zona incerta as a likely route through which the cerebellar signals reach the somatosensory cortex.

Several questions remain. For example, do climbing fibers exert this modulatory effect on cortical plasticity in rewarding or aversive behavioral contexts? The whisker stimulation protocol used in this study was passive, meaning the animal was not engaged in learning a behavior. A rewarding or aversive behavioral context would heavily engage the climbing fiber pathway. Nonetheless, the work of Silbaugh, Koster and Hansel lays the foundations for investigating cerebellar modulation of cortical plasticity, which is likely relevant in both healthy and unhealthy states, such as psychiatric disorders related to cerebellar dysfunction.
